# Dynamical Signatures of Collective Quality Grading in a Social Activity: Attendance to Motion Pictures

**DOI:** 10.1371/journal.pone.0116811

**Published:** 2015-01-22

**Authors:** Juan V. Escobar, Didier Sornette

**Affiliations:** 1 Instituto de Física, Universidad Nacional Autónoma de México, P.O. Box 20-364, México City, 04510, México; 2 Physics Department, Universidad Autónoma Metropolitana-Iztapalapa, México City, 09340, México; 3 ETH Zurich, Department of Management, Technology, and Economics, Switzerland and Swiss Finance Institute, Zürich, Switzerland; National Scientific and Technical Research Council (CONICET)., ARGENTINA

## Abstract

We investigate the laws governing people’s decisions and interactions by studying the collective dynamics of a well-documented social activity for which there exist ample records of the perceived quality: the attendance to movie theaters in the US. We picture the flows of attendance as impulses or “shocks” driven by external factors that in turn can create new cascades of attendances through direct recommendations whose effectiveness depends on the perceived quality of the movies. This corresponds to an epidemic branching model comprised of a decaying exponential function determining the time between cause and action, and a cascade of actions triggered by previous ones. We find that the vast majority of the ~3,500 movies studied fit our model remarkably well. From our results, we are able to translate a subjective concept such as movie quality into a probability of the deriving individual activity, and from it we build concrete quantitative predictions. Our analysis opens up the possibility of understanding other collective dynamics for which the perceived quality or appeal of an action is also known.

## Introduction

Despite the complexity of human interactions, understanding and ultimately predicting individual and collective human behavior would have deep implications in Economics and Psychology not to mention Finance and Advertising [1–5]. To this end, a fruitful approach is based on a parallel that exists in some cases between social and physical systems: when a portion of a social/natural system originally in some sort of equilibrium is driven out of it by an external perturbation (or “shock”), the laws governing its constituting people/particles determine how the information about the shock is spread within the system [1, 6, 7]. Studying the response function to given shocks, the laws behind people´s decisions and interactions can be uncovered. Many aspects of collective social behavior are actually dominated by diffusion-like processes in which the topology of a social network determines how information is transmitted [8, 9]. Dramatic evidence for this can be found in the online activity dynamics during the recent social movements of the so called Twitter revolution [9–12]. Of particular interest is to find the conditions that can bring the system to an “explosive” or “viral” state where information travels quickly and reaches almost the whole network. While government officials may fear a pandemic breakout or a social movement going viral, discovering exactly how to trigger such a state would constitute the wholly grail of advertisement [13, 14]. In this respect, compared to broadcasting, being exposed to direct opinions or recommendations from acquaintances tends to have a much deeper and longer lasting impact [8]. Indeed, word of mouth has been recognized to play a paramount role in turning social movements effervescent [8–12], online videos viral [6], books into best sellers [7], songs into hits [15], and movies into block busters [16, 17] or DVD commercial successes [18]. However, experience tells us that not every recommendation carries the same weight: the better we find a product to be or the more strongly we feel about a social cause, the more enthusiastically will we spread the word about it [19]. Thus, perceived quality is bound to play a fundamental role in the dynamics of social systems.

**Figure 1:**
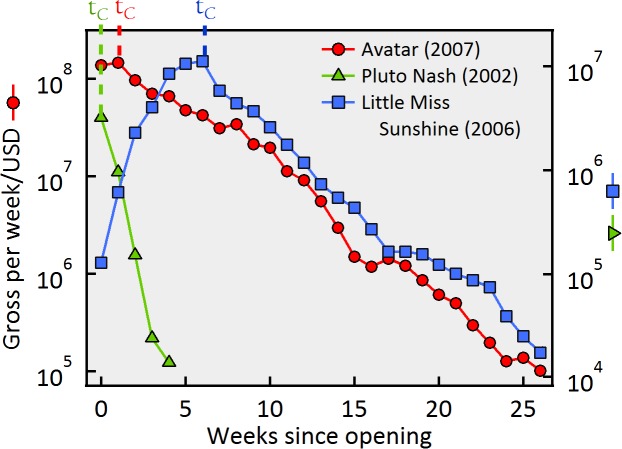
Attendance dynamics for movies representing different shocks and qualities. Weekly gross *vs*. time for *Little Miss Sunshine* (squares, Endogenous), *Avatar* (circles, Exogenous, good movie) and *The adventures of Pluto Nash* (triangles, Exogenous, bad movie).

In this work, we attempt to gain insight into the nature of individual and collective social behavior by investigating the influence that the average perceived quality has on the aggregated dynamics of the attendance to ~3,500 movies in the US. Here, the information being propagated is the perceived quality of a movie, and we make the reasonable assumption that its dynamics is reflected directly in the box-office performance. Indeed, it has been shown that reviews have an effect on people’s decisions to watch a movie [20]. A concrete example of the effect of the popularity on the dynamics of this social system is displayed in Fig. 1, in which we have labeled *t_c_* the number of weeks that have elapsed from the date of the opening to the week of the maximum attendance. In contrast to the worldwide blockbuster *Avatar (2009)*, the attendance to a notorious flop such as *The Adventures of Pluto Nash (2002)* decays sharply after the opening week until it is finally taken out the theaters on week 5.

A characteristic normally used to group similar types of aggregated dynamics into classes is whether a burst of activity is driven by a large external (or Exogenous shock) event, or rather it is the cumulative result of smaller positive factors (or Endogenous shock) [21–25]. In Fig. 1, the time series of *Little Miss Sunshine* is an example of an Endogenous shock in this system, which relied on continuous recommendations from former viewers to create a multiplicative effect that brought the audience to a maximum level many weeks after the opening day. The other two movies shown belong to the Exogenous shocks class, as the peak of attendance occurs on the first or second week. It is expected that the dynamics arising from exogenous and endogenous excitations will present different signatures from which key information about the social system can be extracted and theoretical models be validated.

We show below that the laws behind the relaxation dynamics exemplified in Fig. 1 can be understood, quantified and therefore predicted in terms of the movie’s popularity. In a nutshell, the key concept behind our findings is quite intuitive: the better the movie, the more likely it is that a first-generation viewer will recommend it to her peers, creating an avalanche of attendances that will shape the dynamics of the attendance. The remarkable fact is that this qualitative intuition leads to precise reproducible quantitative predictions. Given that going to the movies is a widespread social activity in the U.S. (in which as many as fifty million people in total can watch a single movie), this activity constitutes an ideal social system to quantify the impact of the perceived quality of a product or the appeal of an action on the dynamics of the spreading of information in human activities.

The reader may question why we need to invoke an agent-to-agent microscopic mechanism describing a kind of epidemic interaction, while perhaps a simple mean-field or representative agent model such as the Bass diffusion model [26] might be sufficient. In this respect, de Vany and Lee [27] have shown the relevance of information cascades in the dynamics of motion picture´s revenue, and have proposed an agent-based model to deal with the rich and intermittent nature of the dynamics of movie attendance. Our message is that the class of epidemic branching models that we use here is actually well-known to be of the “mean-field” class, allowing simple and exact analytical treatment. And to describe the dynamics of social influence, we use the simplest hierarchical decomposition provided by a branching framework that allows one to decompose the dynamics to exhibit important relationships between activity before and after peaks. Such predictions are beyond the reach of the Bass diffusion model or similar models, because they do not distinguish between generations of activity that are deeply associated with the quality of the product and the propensity for propagation in the community.

## Methods

### Dataset and Perceived Quality Function

The initial dataset consists of a collection of time series of the weekly revenue of about 10,000 movies that played in the U.S. during the period from 1970 to 2010 obtained from http://www.boxofficemojo.com/ now owned by the Internet Movie Data Base (IMBD). In order to be able to perform an analysis of statistical significance, we study the post-peak dynamics of the attendance to those 3,469 (~35%) movies that played for at least four consecutive weeks in no less than 50 theaters. This data is complemented with the corresponding audience ratings given by a collection of American letter-system grades *(A, B, C, D, F)* provided by subscribers to this website. We assign to each movie a single normalized grade *G≡ (0.5A´+(7/20)B´+0 C´-(7/20) D´-0.5F´)+0.5*, where the symbols with apostrophes are the normalized number of votes a movie obtained for that particular grading letter. With this definition, *G* always lies between 0 and 1. Applying the simple selection criterion described above ensures that 99% of the movies analyzed have at least 60 ratings, a number that validates the statistically significance of the average normalized grade. Details on data filtering as well as the general trends of the data can be found in S3 Appendix and S7 Appendix respectively. The linear-logarithmic scales of Fig. 1 suggest that the time series of activity *λ*(*t*) of the post-peak weekly gross for each movie can be fitted to a decaying exponential [28] given by:

$$\lambda (t)=\lambda (t_{c})e^{-(t-t_{c})/\tau_{0}}$$ (1)

where *λ*(*t_c_*) is the maximum activity at the peak occurring at time *t_c_* and (1/τ_0_) is the decay rate of total activity for each movie equal to the inverse of their typical lifetime τ_0_ in theatres. An algorithm similar to that used in other studies designed to fit noisy data [6] was employed to fit the time series to equation 1 (see complete methods in S11 Appendix). The data set is in general very well fit to equation 1, with an average R^2^ coefficient of 0.98 (see details on data filtering in S3 Appendix).

### Theoretical Model

**Figure 2:**
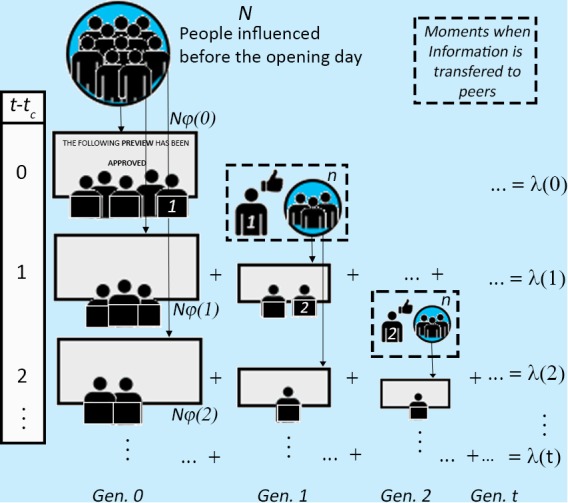
Schematics of the epidemic branching model, eq. 3. Arrows point to the progressive attendance due to the latency effects given by φ(*t*), while the dashed squares shows the moments when previous viewers influence or “infect” new potential viewers that constitute new generations. The total attendance at time *t* (right column) is the sum of the contributions from all previous generations. Only interactions from viewers labeled “1” and “2” are shown here, but all previous viewers influence an average of *n* peers. See S1 Appendix for details.

To describe the dynamics of the attendance, we implement an epidemic branching model with latency effects [6, 7, 19, 29] whereby former viewers influence or “infect” acquaintances to watch the same movie with a probability that depends on its perceived quality. This model is known as the self-excited conditional Poisson process or “Hawkes” process [29], and is composed of two main ingredients. First, it is assumed that former viewers can influence new potential ones within their social network. Once they have already watched it and formed their own opinion, people from this new generation may recommend the movie themselves to their own acquaintances, and so on. This constitutes an epidemic branching process whose end result is the formation of a cascade of attendances characterized by a single parameter *n* called the “branching ratio” (see Fig. 2). The branching ratio is defined as the average number of events triggered in direct lineage by a single mother event. For our case of study, we expect the perceived quality of a movie to be correlated with *n*, as *n* quantifies the propensity for an epidemic of influences to propagate.

The second ingredient of the model incorporates the fact that, once a person has made a decision to perform an action, she will not do it right away, but rather in some future time. This latency effect is characterized by a function that gives the probability that she will perform an action at a time *t*, given that she was influenced or “infected” at a previous time *t_i_*. This function *φ(t—t_i_*) is called the “bare kernel”, or “bare propagator” and is interpreted as the average time between cause and action of a single individual, which may vary depending on the specific human activity. In [30], this distribution is referred to as the “law of procrastination” resulting from an optimization by human agents to maximize the utility derived from their activities. From general priority queuing theory, far from criticality (where criticality is defined by the equality between the rate of incoming tasks and the rate of solving them), one can expect *φ(t—t_i_*) to be an exponential function. Indeed, while in most social systems studied so far [5–7, 19, 31–34], the bare kernel is given by φ(*t*-*t_i_*) ∝ (*t*-*t_i_*)^−(1+*θ*)^ (where *0 < θ* < *1*), here we find that a decaying exponential form gives an excellent description of the activity:

$$\varphi (t-t_{i})=\varepsilon e^{-(t-t_{c})/\tau },$$ (2)

where *0* < 1/τ and ε = *e* ^1/τ^ (1−*e* ^−1/τ^) ensures the normalization of *φ(t*) to sum to unity over the discrete time steps. The exponential form of *φ(t*) implies that our problem lives in the regime where the rate of arrival of new movies is significantly larger than the rate at which most people go to the theatres to watch them, i.e., in general people do not try to maximize the number of movies they watch, and missing a specific one does not have important repercussions. Combining the bare kernel with the branching process, the proposed self-excited model gives the instantaneous rate of attendance at a time *t*:

$$\lambda (t)=S(t)+\sum_{i,t_{i}\leq t}^{t}n_{i}\varphi (t-t_{i})$$ (3)

where *n_i_* is the “fertility” of a previous attendant, defined as the total number of viewers of first generation (i.e. directly influenced by the attendant *i*) that will watch that movie at any future time and who were influenced by that single person *i* who previously saw the movie at time *t_i_*. The term *S*(*t*) embodies all the Exogenous shocks that may include the spontaneous attendances triggered by publicity or by new theaters showing the movie. These external sources are not directly related to previous viewers.

For the particular kernel proposed in equation 2, equation 3 can be solved in closed form for discrete time steps (see S1 Appendix for the derivation) to give:

$$\lambda (t)\propto e^{-\frac{t}{\tau }(1-\theta )},$$ (4)

where

$$\theta (n)\equiv Log{\left(n\left(e^{1/\tau }-1\right)+1\right)}^{\tau }.$$ (5)

**Figure 3:**
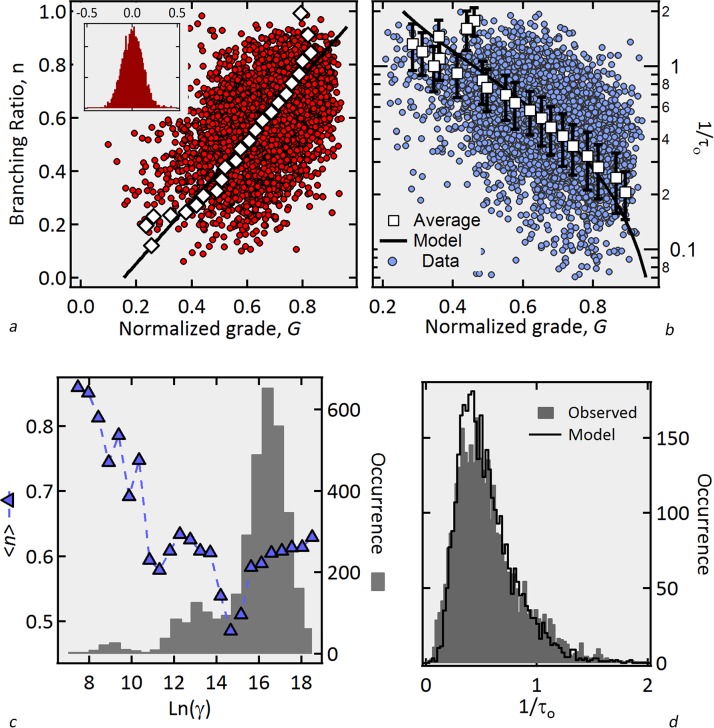
Results: Correlation between the branching ratio *n* and normalized perceived quality grade. a) Observed branching ratio (dots, calculated from eq. 7) as a function of the perceived quality *G*. Solid line corresponds to the axis of symmetry of the data (eq. 8 with α = -49.5^o^ and Δ*y* = 0.118; see S4 Appendix). Squares symbols are the average of *n* around this axis (bin size = 1/35). b) Observed decay rate 1/τ_0_ (circles), prediction curve (line) and averages of the data (squares) as a function of *G*, where error bars are the standard deviation and the constant (1/τ) = 4 was used for all movies. c) Average observed *n vs. Ln*(γ) (triangles) where γ is the maximum revenue for each movie. The histogram gives the number of movies in each bin of the variable *Ln*(γ). d) Distribution of observed (solid bars) and predicted (black line) decay constants obtained from eqs. 5–8. Inset of Fig. 4a is the distribution of deviations around the symmetry axis, with standard deviation equal to 0.11

In the linear-log representations used in Fig. 1, such an exponential law (eq. 4) qualifies as a straight line, which is approximately supported by the data illustrated in Fig. 1. Equation 4 implies that larger values of the branching ratio lead to slower relaxation functions that are nonetheless still exponential, i.e., the branching process renormalizes the bare kernel while keeping the exponential shape of the decay of attendance as a function of time elapsed since the peak. The observed decay rates 1/τ_0_ (like those presented in Fig. 3) are simply related to the branching ratio and the bare kernel as follows:

$$\frac{1}{\tau_{0}}=\frac{1}{\tau }(1-\theta (n)).$$ (6)

From equations 5 and 6, the branching ratio can be solved in terms of the observed decay constant 1/τ_0_ of each movie and the bare kernel decay constant 1/τ, yielding

$$n=\frac{e^{\theta /\tau }-1}{e^{1/\tau }-1}=\frac{e^{\left(1-\frac{\tau }{\tau_{0}}\right)/\tau }-1}{e^{1/\tau }-1}.$$ (7)

## Results and Discussion

### Branching ratio vs. *G*

Assuming that 1/τ is the same constant for all movies as in other social dynamic activities [2, 3], Fig. 3a shows the branching ratio *n* as a function of the perceived quality grade *G* for the whole dataset as obtained with eq. 7, evidencing a significant correlation between these variables. The solid line in Fig. 3a, defined by two constants, *α* and Δ*y* for the whole dataset, is the axis of symmetry of the data around which the mean is zero and the skewness is minimized, i.e., standard deviation is almost constant as a function of *G* (See S4 Appendix). Similar results would have been obtained using principal component analysis. The deviations around this axis are very well described by a Normal function (inset Fig. 3a) with standard deviation equal to 0.11. This axis of symmetry is the best prediction for *n* as a function of *G* and is given by:

$$n_{pred}(G)=GTan(-\alpha )-\frac{\Delta y}{Cos(-\alpha )}.$$ (8)

Inserting *n_Pred_* from equation 8 back into the expression for *θ*(*n*) (eq. 5), we obtain a prediction of the observed decay rate for each movie (eq. 6). In Fig. 3b, we plot these predicted values along with the observed values of 1/τ_0_ and their corresponding averages as a function of *G*. The distribution of the difference between the observed and predicted decay constants are well fit to a Normal distribution with standard deviation of 0.26 (not shown). As a consequence of this good fit, not only the peak (as in refs.[6, 7, 19]) but also the complete distributions of the observed and predicted values of 1/τ_0_ are in good agreement with each other as shown in Fig. 3d. Fig. 3c shows the average branching ratio <*n*> as a function of *Ln*(γ), where γ is defined as the maximum revenue for each movie, (i.e. γ≡*λ*(*t_c_*)). We observe that *n* is a decreasing function of *Ln*(γ) for *Ln*(γ)< = 15.5 after which point it increases again. Since a larger *n* implies smaller 1/τ_0_, this figure means that the larger the audience on the opening week, the easier the information will be transmitted about the quality of big production movies (*Ln*(γ)>15.5).

**Figure 4:**
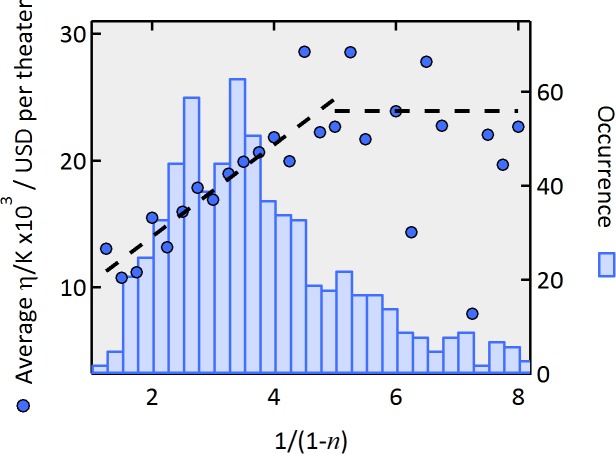
Independent validation of the epidemic branching model. Average accumulated weekly revenue *η* as a function of 1/(1-*n*). Dashed diagonal line is the best fit to a linear relation in the range [1, 5], which validates the epidemic branching model. Dashed horizontal line represents the average of *η* in the range [5, 8]. The blue bars quantify the occurrence number in each bin.

Even though the correlation shown in Fig. 3a is clearly present, correlation does not necessarily imply causation. The question arises whether what we interpret as a rising branching ratio resulting from an avalanche of recommendations and attendances could in fact be due to some other mechanism that is associated with the perceived quality. To further validate our interpretation of these results, we turn our attention to the accumulated weekly revenue up to the week in which the maximum activity is attained for the whole Endogenous class. This accumulated revenue is henceforth denoted by *η*. While the branching ratio *n* is calculated by fitting the data after the time *t_c_* of the peak of activity, we stress that *η* is independently calculated using data up to *t_c_*. As we show below, our cascade model predicts that *η* should be proportional to 1/(1-*n*). The independent estimation of these two variables and their proportionality shown in Fig. 4 thus serves as a remarkable independent evidence of the validity of our model. We now explain the reasoning in details.

**Figure 5:**
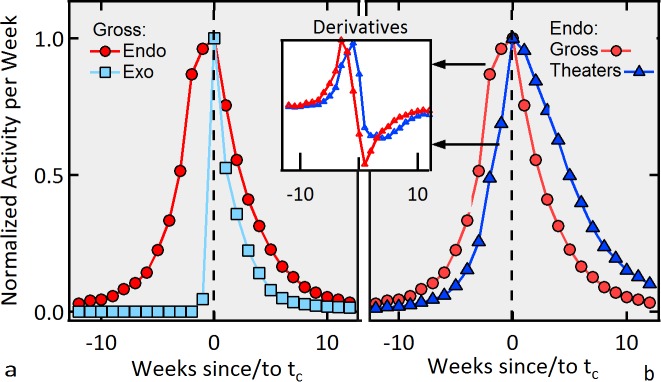
Non-symmetric precursory growth. a) Normalized accumulated gross per week as a function of time, centered on the day of the maximum activity for Endogenous (circles) and Exogenous (squares). b) Normalized accumulated gross per week (circles) and theaters (triangles) for the Endogenous class. Inset) Corresponding time derivatives in arbitrary units *vs*. time. The peak for the activity for the Endogenous class is non-symmetric due to the new external shocks brought by the theaters that open up once a movie proves itself successful. Note that the derivative of the normalized number of theatres playing a given Endogenous movie lags by about 2–4 weeks the derivative of the normalized gross per week.

Consider a small production movie played in *κ_0_* theaters on the opening week with initial audience *m*_0_ proportional to the initial activity *S_0_ = λ*(0). By definition, in our avalanche model, every single attendant influences an average of *n* new ones to watch that movie. If such movie was allowed to play indefinitely, the total number of viewers influenced by the *m*_0_ initial ones would be proportional to *S_0_*(1+*n*+*n* ^2^+*n* ^3^+…) = *S_0_*/(1-*n*). On the other hand, a larger number of movie theaters samples geographically a correspondingly larger number of potential movie goers. Actually, for the Endogenous class, the number of theaters grows in time (Fig. 5b) up to a maximum reached at *t = t_c_*, and each one of these *κ_t_* new theaters may induce a corresponding activity *S_i_* that does not derive from any personal recommendation. These “external sources” are contemplated by our general equation, eq. 3 and will in turn bring a new cascade of activity each with total revenue *S_0_*/(1-*n*), should the movie play indefinitely. If, as a first approach, we assume *S_t_* to be proportional to the corresponding number *κ_t_* of new theatres (*S_i_* = *ακ_i_* where *α* is a constant), then the total revenue *η* up to t = *t_c_* can be approximated as:

$$\eta \equiv \sum_{t=0}^{t_{c}}\lambda (t)\approx S_{0}\sum_{t=0}^{\infty }n^{t}+S_{1}\sum_{t=1}^{\infty }n^{t-1}+\ldots +S_{t_{c}}\sum_{t=t_{c}}^{\infty }n^{t-t_{c}}=\frac{\left(S_{0}+S_{1}+\ldots S_{t_{c}}\right)}{\left(1-n\right)}=\frac{\alpha K}{\left(1-n\right)}$$ (9)

where *K = κ_0_+κ_1_+…+κ_tc_*. Note that the sum of the new theaters is actually equal to the number of theaters playing that movie at *t = t_c_*. Now, if our epidemic branching model is correct, then *η*/*K* should be proportional to 1/(1-*n*). This is shown to be the case in Fig. 4 for values of 1/(1-*n*) in the range [1, 5] (i.e. for *n* in the range [0, 0.8]), where the vast majority of the data reside. Furthermore, the infinite limit approximation used in eq. 9 gives a better description of *η* when *n* is closer to zero (1/(1-*n*) between 1 and 5), than when *n* is closer to 1 (for 1/(1-*n*)>5), because the formula is less sensitive to finite size effects when only a few generations are involved. For large values of 1/(1-*n*), a truncation due to several finite size effects is expected, including the finite number of theatres, movie-goers and limited time a movie is presented. This rationalizes the saturation observed in Fig. 4 for 1/(1-*n*) > 5.

A null-hypothesis could be stated according to which the decay constant measured after *t_c_* is not related of the total attendance measured before *t_c_*,, excluding our proposed mechanism in terms of the avalanches mechanism that are claimed to relate the attendance dynamics both before and after *t_c_*. As we have just shown, Fig. 4 disproves this null-hypothesis. A second null-hypothesis could be that the correlation found between *n* and *G* of Fig. 3a is a spurious result arising from pure chance. To disprove this null-hypothesis, we randomly shuffle the grades for all movies and calculate the Pearson´s correlation coefficient *r* between the sets given by *n* and *G_random._*. We repeat this method for 10,000 different sets of *G_random_* and obtain the distribution of *r*, which is very well fitted to a Normal distribution with standard deviation σ = 0.017 (not shown). In contrast, the actual sets of *n* and *G* give *r* = 0.4. Thus, the probability that we obtained such a correlation between *n* and *G* is essentially zero, i.e. the “*p*-value” for the null hypothesis to be true is essentially zero.

We recognize that the social system under study is extremely complex, in which many factors can in principle determine the success of a movie. Some of these factors may include competition, finiteness of resources for advertising, imitation *vs*. resistance *vs*. resilience in the public, or fashion waves. While investigating in detail the effects of each one of these factors would be of great interest, such a task lies outside of the scope of this article. However, as an example, in S10 Appendix we briefly explore how the dynamics of the attendance is affected by the competition between movies.

### Endogenous shocks mechanism

**Figure 6:**
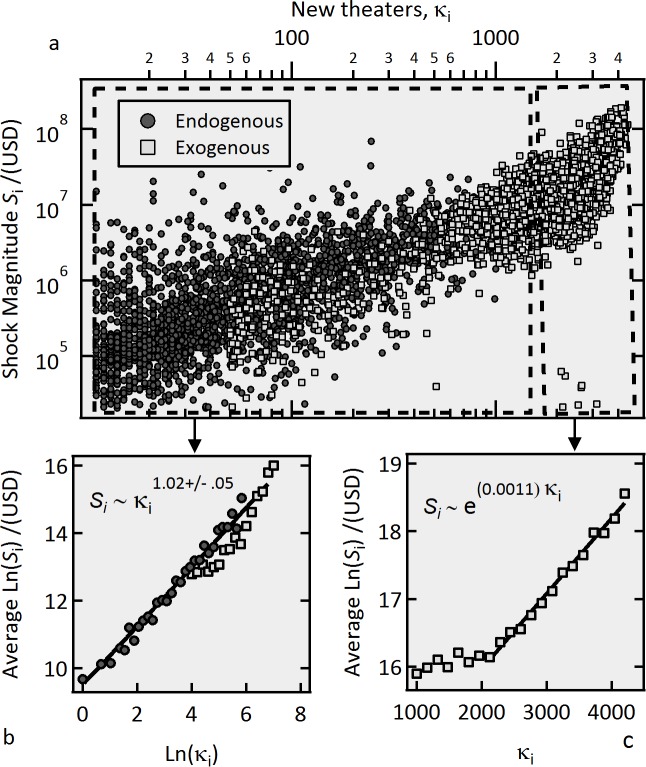
External sources of new shocks. a) Magnitude of the induced shocks *S_i_* *vs*. number of new theaters κ_*i*_. The red circles depict *S_i_* as calculated with equation 9 for the precursors’ activity of the whole Endogenous class. In the calculation of *S_i_*, the activity from previous generations was properly subtracted (see S2 Appendix). The squares represent the maximum activity γ_*i*_ *vs*. the number of theaters that played the movies on their opening week for the Exogenous class. (b) Average *Ln*(*S_i_*) as a function of *Ln*(κ_*i*_) for the Endogenous precursors, showing a linear dependence. (c) Average *Ln*(*S_i_*) *vs*. κ_*i*_ for the Exogenous class. For κ_*i*_ >2000 the dependence becomes exponential.

Endogenous shocks are usually the cumulative result of small positive perturbations that, in the case of a power law kernel, renormalize the activity yielding a slower decay [6, 7, 19, 35]. A second important feature arising from the action of these small perturbations is that the activity is symmetric around the peak. Note in Fig. 1, however, that this is not the case for *Little Miss Sunshine*, nor is it in general for the rest of the Endogenous movies as can be appreciated in the accumulated normalized activity displayed in Fig. 5a. These facts suggest that, in this social system, there exists a different mechanism responsible for the pre-peak growth we find, as anticipated by the analysis performed in the previous section. The key to finding this mechanism can be determined from an important difference between the attendance to movies and other social activities such as watching videos or buying books online: the accessibility of the product. While the latter two activities can be performed at all times (assuming the book is not off-print), in the case of movies, the specific film in which we are interested must be playing nearby our usual whereabouts for us to go and watch it. It is then reasonable to assume that the number of theaters playing a movie at different times during the lifetime of a movie will be a factor in determining the dynamics of the attendance. To test this hypothesis, in Fig. 5b, we show both the normalized accumulated gross per week and number of theaters for the whole Endogenous class. One can observe that, once a movie begins to perform well (according to some standard), new theaters will decide to play that movie in an effort to profit from this proven success, i.e., supply adaptation is dynamic [19]. The inset of Fig. 5 displays the corresponding time derivatives. Note how, even though the response from the supply side (the available theaters) is slow at the beginning, eventually it becomes faster than the growth rate of the revenue itself until a maximum number of theaters is reached. At this point, when no more theaters open up, the activity of the attendance reaches its maximum and the system relaxes thereafter following an exponential decay with constant (1/τ_0_). If this picture is correct, then new theaters serve as sources of new external shocks *S*(*t*), as contemplated in our general equation 3 for the activity. Let the time series of these new theaters be {κ_*i*_}, which is also known for each movie. To obtain the series of new shocks {*S_i_*}, given the activity *λ*(t), we assume that every new generation *g* of movie goers evolves in time following eq. 1 with the same 1/τ_0_ observed after the peak for that Endogenous movie. Then, the external sources can be obtained recursively as:

$$S(t)\equiv S_{t}(0)=\lambda (t)-\sum_{g=0}^{t-1}S_{g}(t-g)$$ (10)

where *S_g_* (*t*-*g*) = *S*_0_ (0)^*e*-(*t*-*g*)/τ0^, and *S*_0_ (0) ≡ λ(0). For a given Endogenous movie, the time series *S_i_* is obtained using equation 10 assuming the measured 1/τ_0_ for that movie (Fig. 3b) applies to every new generation (see S2 Appendix for the derivation of eq. 10, and S8 Appendix for an example of its application). To investigate the response function of the system, we analyze the relation between *κ_i_* and the corresponding *S_i_* for the whole set of precursors of the Endogenous shocks (3586 such events were extracted) in Fig. 6. The circles in Fig. 6a and the corresponding averages shown in Fig. 6b strongly suggest there exists a power law relationship between the number of new theaters and the magnitude of the shocks the produce. We find a power law exponent of 1.01 +/- 0.05, which corresponds to a linear relation, i.e. *S_i_* ∝ *k_i_*, as we had anticipated in the analysis of Fig. 4. We realize that the proposed proportionality between *κ_i_* and *S_i_* can only hold on average, because it is the eventual weakening of the response (or in mathematical terms, the eventual reduction of the susceptibility) from the public to a given “impulse” what brings the activity to a maximum. In that respect, further analyses of the time evolution of the average *S_i_*/*κ_i_* would provide insights about the response function of this social system.

We have postulated that the dynamics of the relaxation in this social system is universally given by eqs. 3–5, regardless of the type of shock. It is then interesting to verify if there exists a difference in the excitation process between Endogenous and Exogenous shocks. To this end, we perform the equivalent *S_i_* *vs. κ_i_* analysis now with the set of Exogenous initial shocks. Noting that, in this case, there are no precursors, we take as single sources of excitation the number of theaters *κ_i_* from the opening week and the corresponding maximum gross per week γ_*i*_ as the magnitude of the shock (squares, Fig. 6a).

Fig. 6b shows that the events from the Exogenous shocks extend the power law trend of the Endogenous precursors up to a limit *κ_i_* = 2000. In other words, new theaters serve as linear sources of new shocks irrespective of whether a movie belongs to the Endogenous or the Exogenous class. Nevertheless, for larger events than those accessible by Endogenous shocks, (*κ_i_*> 2000), the log-linear graph on Fig. 6c suggests that the relation between *κ_i_* and *S_i_* transitions to an exponential function, signaling a change has taken place to an exponential regime. Note in the y-axis of Fig. 6c that this transition happens to movies with *Ln*(γ)>16.2. This corresponds roughly to the same threshold we find in Fig. 3c after which the average branching ratio becomes an increasing function of *Ln*(γ). These two pieces of evidence suggest that there exists a critical level of connectivity [10, 36] in the social network above which the interaction between people becomes non-linear and as a result, much stronger. Taking the average ticket price to be $10, this threshold can be estimated to be of the order of (*e* ^16.2^/10) ≈ 1 million people.

### Total revenue *vs. G*

Even though the strong linear correlation that we find between the perceived quality and the branching ratio is quite revealing, what is utterly relevant for the movie industry is the impact that this relation has on the total revenue. As it turns out, the effect of the perceived quality on the revenue is also non-linear. To illustrate this, take two of the movies analyzed in Fig. 1, *Little Miss Sunshine* and *The adventures of Pluto Nash* that presented roughly the same maximum weekly revenue. While the former ended up making $60M, the later made only 4.4M at the box office. Indeed, *Little Miss Sunshine* was an Endogenous smash-hit for which every former viewers brought to the theaters an average of *n*(*G* = 87%) *≈* 0.83) new ones, whereas those who saw *Pluto Nash* influenced on average only *n*(*G* = 20%) *≈* 0.05) new viewers. Note that even though the ratio of their grades is (0.87/0.20) *≈* 4, the ratio of their earnings is almost 14. This non-linear effect can be understood by writing explicitly the approximate total revenue (after the maximum activity was reached) assuming the movies are exhibited indefinitely in theaters:

TotalRevenue≈γ∑t=tc∞e−(t−tc)/τ0=γ(11−e−1/τ0)=γ(11−e−(1−θ)/τ)≈γ(11−e−(1−n)/τ), (11)

where *n* = *n*(*G*) is a function of the perceived quality given by equation 8, and the approximation *θ≈ n* was used. Recall that γ is the peak revenue of the corresponding movie. The error obtained by using the infinity limit in equation 11 is actually less than 5% (see S5 Appendix). According to equation 11, as the branching ratio tends to 1, the revenue becomes infinite. However, the relation between *n* and *G* (eq. 8) sets a limit on the maximum *n* attainable. With the parameters used to fit the data in Fig. 3 (*α* = -49.5^o^ and Δy = 0.11), then *n*(*G* = 100%) = 0.986, which in turn implies that the total revenue is at most equal to 82γ, but may be as small as 1.08γ for *n*(G = 20%) = 0.05.

**Figure 7:**
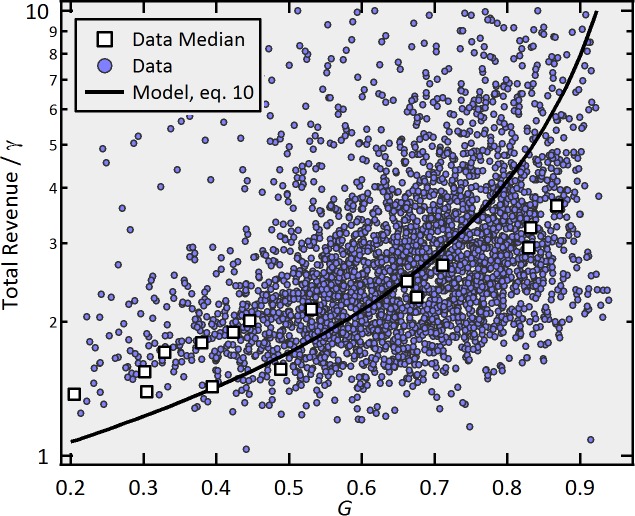
Revenue ratio vs. *G*. Total revenue divided by the peak revenue γ for all movies belonging to the Exogenous class (dots) and the prediction of the model (line, eq. 10). The total revenue is the actual profit of these movies as found on the website.

Fig. 7 shows the total revenue earned after *t_c_* divided by the corresponding peak revenue γ as a function of *G* for each movie belonging to the Exogenous class and their median values, along with the prediction of our theoretical model. Through eq. 11 and Fig. 7, our model explains why it is so important for the film industry that audiences like and recommend the movies it produces: since the box office earnings on the first week is of the order of *e* ^16.5^ ≈ 15 million dollars (see S7 Appendix), a “must see” movie (*G* = 90%) can make (11.9 × 15 million) = 178 million dollars while a “don´t watch it” one (*G* = 20%) will only make (1.08 × 15 million) = 16.2 million, even if both movies were equally successful during the opening week. This analysis becomes even more relevant if we consider that the average movie costs about $100 million dollars to produce [37]. These results help explain the conclusions of other studies that have found that launching advertising campaigns after the movie has already opened does not have a strong impact on the final revenue [20]. Broadcasting can only go so far.

## Conclusions

We have shown how “perceived movie quality” translates into a probability of watching that movie and the power of direct connections to spread information within social networks. While this relation is linear, its effect on the total number of people involved in this activity is exponential.

Further studies regarding the transition we find in the response of the system to external shocks from linear to exponential (Fig. 6) could give information about the topology of the network. In turn, this could help predict the dynamics of other social processes in which over 1 million people serve as the nucleation threshold for global information propagation. These activities may range from picking candidates in governmental or presidential elections and supporting humanitarian causes, to starting a social movement or buying the newest smart phone.

The results presented in this work open up the possibility of understanding and predicting the dynamics of a wide range of social systems for which the perceived quality is recorded. The advent of the internet has made it as easy as pressing a key to express our assessment of millions of products, activities, social causes, political views, and many others, in a society that is addicted to ranking [38] tagging [39] and betting [40]. Similar methods to the ones we have applied here could provide concrete quantifiable metrics of peoples opinion’s concerning readily available or future social activities or commercial products.

## Supporting Information

S1 Appendix Hawkes process with exponential kernel: Response function for Exogenous Shocks. (PDF)

S2 Appendix New theaters as sources of Exogenous shocks: precursors for Endogenous shocks. (PDF)

S3 Appendix On data filtering. (PDF)

S4 Appendix Finding the axis of symmetry. (PDF)

S5 Appendix Total revenue vs. Grade. (PDF)

S6 Appendix Correlation between the normalized grade *G* used in the present paper, and the “Audience Average Rating” obtained from the website www.rottentomatoes.com. (PDF)

S7 Appendix General Trends of the data. (PDF)

S8 Appendix An example of how new theaters serve as a source of external shocks. (PDF)

S9 Appendix Note on medians. (PDF)

S10 Appendix Correlation between then number of movies playing, and *n* and *n/G*. (PDF)

S11 Appendix Methods. (PDF)
