# Supplementary material for: Dynamical Signatures of Collective Quality Grading in a Social Activity: Attendance to Motion Pictures
Source: PLoS One. 2015 Jan 22;10(1):e0116811. doi: 10.1371/journal.pone.0116811 (PMC4303319; doi:10.1371/journal.pone.0116811)
Supplement: S1 Appendix — (PDF) [file pone.0116811.s001.pdf]

SUPPORTING INFORMATION for the paper:

Dynamical signatures of collective quality grading in a social activity: attendance to motion pictures

by Juan V. Escobar & Didier Sornette

S1 Appendix: Hawkes process with exponential kernel: Response function for Exogenous Shocks.

S1.A Discrete time solution of equation 3 (epidemic branching process with a decaying exponential bare kernel)

There are two ways to obtain the total activity as a function of time. The first one orders the events in delay and triggering as follows.

First derivation of the total activity as a function of time

Delay: Assume first that branching processes are absent and only latency effects exist. Suppose that news about a movie are released at time $t = t_r$, which will open at time $t_c \equiv (t_r + 1)$. Then, a first generation of N spontaneous viewers will eventually watch the movie (constituting the "immigrant" generation to use the language of branching process), and they will do so with a probability given by the exponential bare kernel, $\phi(t - t_r)$. In other words, the assistance to theaters by the population influenced (or infected) at time t_r shows a distribution of delays quantified by $\phi(t - t_r)$ given by expression (2). This activity will begin at time $t = (t_r + 1)$, and is given by

$$\lambda_d(t - t_r) = N\phi(t - t_r) = N\, e^{-\frac{(t-t_r)}{\tau}} e^{\frac{1}{\tau}} \left(1 - e^{-\frac{1}{\tau}}\right). \qquad \text{(S1)}$$

Normalization of the bare kernel ensures that $\sum_{t=t_r+1}^{\infty} \lambda_d(t - t_r) = N$. This exponential bare kernel has the unique property that the delayed activity at time t is proportional to the delayed activity at time $(t - 1)$, or

$$\lambda_d(t - t_r) = \alpha\, \lambda_d(t - t_r - 1), \qquad \text{(S2)}$$

where $\alpha \equiv e^{-\frac{1}{\tau}}$. It is worth pointing out that this property is not shared by the power law bare kernel used in other works mentioned in the main text. Eq. S2 allows us to re-write the delayed activity for this generation at any future time simply as

$$\lambda_d(t - t_r) = \alpha^{t-t_r-1}\, \lambda_d(1), \qquad \text{(S3)}$$

for $t > t_r + 1$, where

$$\lambda_d(1) = N \left(1 - e^{-\frac{1}{\tau}}\right). \qquad \text{(S4)}$$

In particular, the contribution from the delayed activity at $t = t_r + 2$ is

$$\lambda_d(2) = \alpha\, \lambda_d(1). \qquad \text{(S5)}$$

Triggering: Now consider the new spectators that will be influenced to watch a movie, keeping in mind that only those who have already seen a movie can recommend it. We first isolate only those events triggered by the immigrant generation, $\lambda_d(1)$. These new events will constitute the first generation of viewers and correspond to the start of the epidemic portion of the Hawkes process. Assume that a total of N' people are persuaded by previous viewers at time $t = (t_r + 1)$ to watch the movie. By definition, the branching ratio is equal to $n = N'/\lambda_d(1)$. Once again, the newly influenced people will not perform the activity all at once, but rather will do it

following the same probability function given by the bare kernel (eq. S1), with N replaced by $N' = (n\,\lambda_d(1))$, and starting at time $t = (t_r + 2)$. The time series of the first generation (or triggered events, $\lambda_t(t - t_r)$) will then be given by the equivalent of equation S2:

$$\lambda_t(t - t_r) = \alpha\,\lambda_t(t - t_r - 1), \quad \text{(S6)}$$

for $t > (t_r + 2)$. The first element of this series (equivalent to Eq. S4 for the delayed contribution) is $\lambda_t(2)$ = $(n\,\lambda_d(1))\ \left(1 - e^{-\frac{1}{\tau}}\right)$, or

$$\lambda_t(2) = \beta\,\lambda_d(1)\,, \quad \text{(S7)}$$

where $\beta \equiv n\ \left(1 - e^{-\frac{1}{\tau}}\right)$. Thus, at time $t = (t_r + 2)$ there are going to be two contributions to the total activity: one from delay effects (eq. S5) and another one from triggering ones (eq. S7). However, both terms are proportional to the previous activity $\lambda_d(1)$ so that it can be factored as follows:

$$\lambda_{Total}(2) = \lambda_t(2) + \lambda_d(2) = \alpha\,\lambda_d(1) + \beta\,\lambda_d(1) = (\alpha\ + \beta)\lambda_d(1). \quad \text{(S8)}$$

Note further that both terms of eq. S8 will evolve in time following eqs. S6 and S2 (i.e., applying the operator α on each term), so that the delayed contributions at time $t = (t_r + 3)$ are:

$$\lambda_d(3) = \alpha\,\alpha\,\lambda_d(1) + \alpha\,\beta\,\lambda_d(1) = \ \alpha\,(\alpha\ + \beta)\lambda_d(1). \quad \text{(S9)}$$

On the other hand, the total activity at $t = (t_r + 2)$, $\lambda_{Total}(2)$, becomes the source of newly triggered events beginning at time $t = (t_r + 3)$ (the equivalent to equation S7). Then, the first element of the second generation is:

$$\lambda_t(3) = \beta\,\lambda_{Total}(2) = \beta(\alpha\ + \beta)\,\lambda_d(1). \quad \text{(S10)}$$

The total activity at $(t - t_r) = 3$ is the sum of these two contributions, eqs. S10 and S9,

$$\lambda_{Total}(3) = \lambda_t(3) + \lambda_d(3) = \ (\alpha\ + \beta)^2\,\lambda_d(1). \quad \text{(S11)}$$

Note that once again, the initial activity $\lambda_d(1)$ is factorized in the expression for the total activity at this time. It is straightforward to prove by induction that this will be the case for an arbitrary time, so that the total activity is given by

$$\lambda_{Total}(t - t_r) = (\alpha\ + \beta)^{(t - t_r) - 1}\,\lambda_d(1), \quad \text{(S12)}$$

for $t \geq 2$, where $\lambda_d(1) = N\ \left(1 - e^{-\frac{1}{\tau}}\right)$ is the very first activity that is observed, i.e., the activity on the opening week at $t = t_r + 1 \equiv t_c$. For our analysis, it is more convenient to write equation (S12) directly in terms of t_c instead of t_r because, as we define in the main text of the paper, t_c is the week of the maximum activity for both Endogenous and Exogenous shocks. The total activity as a function of $(t - t_c)$ defined as $\lambda'_{Total}(t - t_c) \equiv \lambda_{Total}(t - t_r + 1)$ is then

$$\lambda'_{Total}(t - t_c) = (\alpha\ + \beta)^{(t - t_c)}\,\lambda'_{Total}(0) = \lambda'_{Total}(0)\ \left(e^{-\frac{1}{\tau}} + n\ \left(1 - e^{-\frac{1}{\tau}}\right)\right)^{(t - t_c)}, \quad \text{(S13)}$$

for $t \geq t_c$, where $\lambda'_{Total}(0) = N\ \left(1 - e^{-\frac{1}{\tau}}\right)$. After some algebraic manipulation eq. (S13) can be re-written in exponential form as

$$\lambda'_{Total}(t-t_c) = \lambda'_{Total}(0)\mathrm{e}^{-\frac{(t-t_C)}{\tau}(1-\theta)}\ , \qquad (1,S14)$$

where

$$\theta \equiv\ \tau\, Ln\, \left\{n\left(e^{\frac{1}{\tau}}-1\right)+1\right\}. \qquad (S15)$$

Note that $\lambda'_{Total}(0)$ is nothing but the maximum activity γ as it was defined in the main text of the paper. Note too that $\theta = 0$ when $n = 0$, and for small n, we have $\theta = n\tau\ \left(e^{\frac{1}{\tau}}-1\right)$, which gives $\theta \approx n$ for small $1/\tau$. Thus, the activity is given always by a decaying exponential whose decay constant is renormalized when the branching ratio is different than zero, i.e., when there is some multiplication effect due to cascades of recommendations. The introduced quantities α and β can be interpreted as linear operators whose sum acts as a (discrete) time-evolution operator when applied to an element of the total activity, in which α acts as the "delay" operator and β as a "trigger" one. In this way, we can lose track of the origin of time t_r in the calculation of the activity because $(\alpha+\beta)$ is applied directly to the immediate previous activity (eq. S2), i.e., we have disentangled the process.

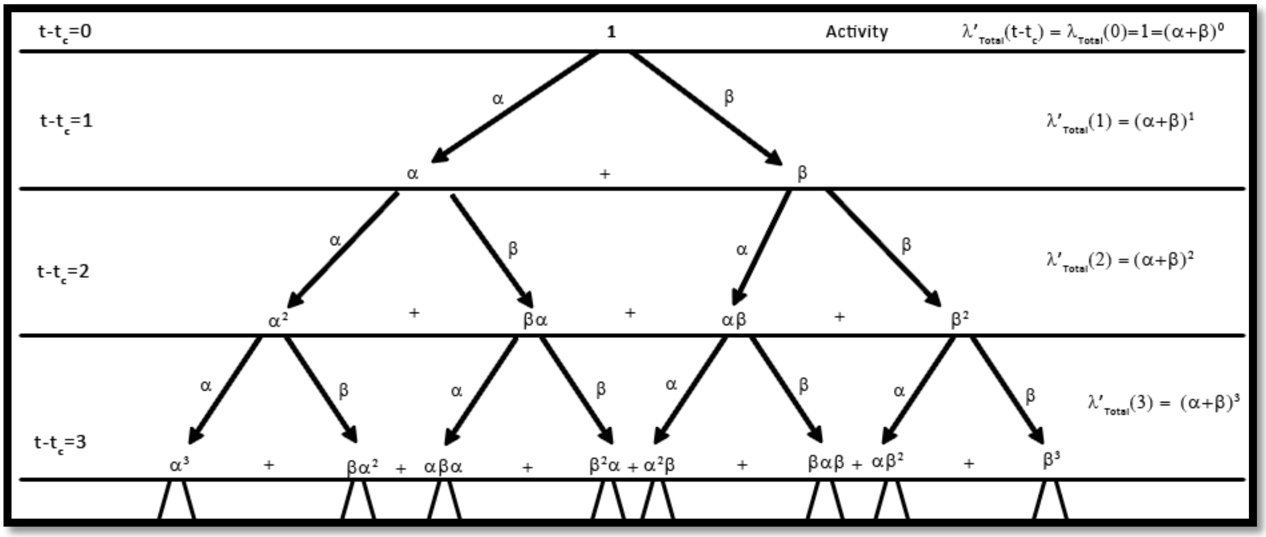

Figure S1. Explicit Branching process diagram. The total activity (right column) at a time $(\boldsymbol{t}-\boldsymbol{t_c})$ (left column) is obtained by applying the operator $(\boldsymbol{\alpha}+\boldsymbol{\beta})$ to the elements of the total activity at the immediate previous time $(\boldsymbol{t}-\boldsymbol{t_c}-\mathbf{1})$. Every term of the binomial expansion (whose sum yields the total activity) keeps track of its own origin in terms of the generation to which it belongs. Note that the activity has been normalized without loss of generality so that $\boldsymbol{\lambda'_{Total}(0)=1}$ to make the branching process clearer.

Figure S1 shows explicitly how successive application of $(\alpha+\beta)$ generates a tree that recovers all the events of the activity and also that this algebraic mapping keeps track of the origin of the contributions of the total activity at any arbitrary time. Without loss of generality, assume that the activity at time $t = t_c$ is $\lambda'_{Total}(0) = 1$. Applying $(\alpha+\beta)$ at every time step is graphically equivalent to adding two bifurcating arrows: an arrow pointing to the left multiplies the element of the previous activity by α, while an arrow pointing to the right multiplies it by β. The total activity at that time is the sum of the resulting elements (right column). Using this tree, the algebraic form of the elements of the activity at a certain time can be mapped into the origin of the elements as follows. Reading from left to right, the power of α gives the number of weeks since the closest branching process took place, which in turn signals the creation of a new generation. The trail that a new generation is created is precisely the operator β, and the power of $\beta's$ gives the generation to which an element belongs. Take for example the elements that constitute the activity at time $(t-t_c) = 2$:

α^2: This is the second delayed element of a process that began at time $(t - t_c) = 0$. Because there is no β present, this element belongs to generation zero.
αβ : This is the first delayed element of a branching process that began at time $(t - t_c) = 1$. Thus, this element belongs to generation one (exponent equal to 1 of β).
βα: This is the first branching element arising from the first delayed element of a process that began at time $(t - t_c) = 0$. This element belongs to generation 1 because of the value 1 of the exponent of β.
β^2: This is the first element of a branching process in turn arising from the first element of a branching process that began at $(t - t_c) = 1$. Because there are two powers of β, this element belongs to generation two,

and so on. Thus, the specific algebraic representation of the elements of the expansion can keep track of their origin.

S1.B Second derivation of the total activity as a function of time

An alternative way of obtaining the total activity as a function of time is to use an ordering in time as follows. Define $\varepsilon \equiv e^{1/\tau}\left(1 - e^{-\frac{1}{\tau}}\right)$ so that the bare kernel is written as $\phi(t) = \varepsilon\, e^{-t/\tau}$. The total activity $\lambda_{Total}(t)$ at time t (in discrete times) due to a single mother event can be written as

$$\lambda_{Total}(t) = \{n\,\phi(t)\} + \{n\,\phi(1)\,n\,\phi(t-1) + n\,\phi(2)\,n\,\phi(t-2) + \cdots + n\,\phi(t-1)\,n\,\phi(1)\} + \{two\ intermediate\ events\} + \{three\ intermeidate\ events\} + \ \ldots + \{k\ intermediate\ events\} + \cdots \quad \text{(S16)}$$

where $\{n\,\phi(t)\}$ is the direct triggering of an event at time t from the mother event, the second bracket gives the cases where the activity at time t is due to one triggered event at some intermediate time i, which then triggers an event directly at time t, the bracket named {two intermediate events} involves two intermediate events and so on. The "two intermediate events" bracket can be seen to sum up to $(n\,\phi(t)\,n\,(t-1)\,\varepsilon)$. Note that the term $(t-1)$ is the combinatorial factor of choose 1 item among $(t-1)$, or $C(t-1,1)$. Likewise, we can see by inspection that the "three intermediate events" bracket sums up to $(n\,\phi(t)\,C(t-1,2)\,(n\varepsilon)^2)$, where $C(t-1,2)$ is the combinatorial factor of choose 2 items among $(t-1)$, removing the order influence. Following this reasoning, the "k intermediate events" bracket sums up to $(n\,\phi(t)\,C(t-1,k)\,(n\varepsilon)^k)$ where $C(t-1,k)$ is the combinatorial factor of choose k items among $(t-1)$ and is explicitly given by $\frac{(t-1)!}{(t-1-k)!k!}$.

Using these expressions, the total activity can be written as

$$\lambda_{Total}(t) = n\,\phi(t)\,\{1 + C(t-1,1)\,n\varepsilon + C(t-1,2)\,(n\varepsilon)^2 + \cdots + C(t-1,k)\,(n\varepsilon)^k + \cdots\}. \quad \text{(S17)}$$

We recognize the terms inside the brackets as the series expansion of $(1+n\varepsilon)^{t-1}$, so that

$$\lambda(t) = n\,\phi(t)\,(1+n\varepsilon)^{t-1}. \quad \text{(S18)}$$

Substituting the expressions for $\phi(t)$ and ε, and after some algebraic manipulation, equation (S18) can be written as

$$\lambda_{Total}(t) = n\left(1 - e^{-\frac{1}{\tau}}\right)\left(e^{\frac{-1}{\tau}} + n\left(1 - e^{-\frac{1}{\tau}}\right)\right)^{t-1} = n\left(1 - e^{-\frac{1}{\tau}}\right)(\alpha + \beta)^{t-1} = \lambda_{Total}(t)(\alpha + \beta)^{t-1}$$

(S19)

which is precisely equation S12 derived above with a different method with $t_r = 0$ and a different initial value $\lambda_{Total}(1)$ because in this derivation we consider a single mother event.
