# Supplementary material for: Dynamical Signatures of Collective Quality Grading in a Social Activity: Attendance to Motion Pictures
Source: PLoS One. 2015 Jan 22;10(1):e0116811. doi: 10.1371/journal.pone.0116811 (PMC4303319; doi:10.1371/journal.pone.0116811)
Supplement: S2 Appendix — (PDF) [file pone.0116811.s002.pdf]

SUPPORTING INFORMATION for the paper:

Dynamical signatures of collective quality grading in a social activity: attendance to motion pictures

by Juan V. Escobar & Didier Sornette

S2 Appendix: New theaters as sources of Exogenous shocks: precursors for Endogenous shocks.

The events depicted by circles in figure 6a correspond to the instantaneous new attendance (or Exogenous shocks) as a function of the number of new theaters that play a movie (like the positive bars in the example of figure S10). In this section, we derive eq. 10, which is used to obtain the data for the Endogenous precursor's activity of figure 6.

All the information we have is the total attendance per week, $\lambda(t-t_c)$, and the observed decay constant $1/\tau_0$ for each movie (measured after the activity peak for Endogenous movies). The key to obtain the contribution from the new shocks is to assume that any new generation g will evolve in time following eq. 1, i.e., with the same decay constant $1/\tau_0$ as follows.

Let $S_g(t-t_c-g)$ be the contribution of the gth generation at time t to the total activity. The first term or every new generation ($S_g(0)$) corresponds to an Exogenous contribution of our master equation (3). In order to calculate these contributions, consider first the total activity $\lambda(0)$ at $t=t_c$. This activity is composed only of <u>zeroth</u> generation viewers ($g=0$), and therefore $\lambda(0)=S_0(0)$.

Recall that the subscript 0 refers to the fact that the element on the right hand side belongs to the zeroth generation. Now, $S_0(0)$ is the first element of a population that will attend the theater according to equation 1 of the main text:

$$S_0(t-t_c)=S_0(0)e^{-(t-t_c)/\tau_0} \tag{S20}$$

In fact, this equation holds in general for any generation g:

$$S_g(t-t_c-g)=S_g(0)e^{-(t-t_c-g)/\tau_0} \tag{S21}$$

In order to obtain the first element of the <u>first</u> generation ($g=1$) at time $(t-t_c)=1$, we need to subtract the first element of the zeroth generation to the total activity. Explicitly:

$$\lambda(1)=S_0(1)+S_1(0), \tag{S22}$$

$$S_1(0)=\lambda(1)-S_0(1), \tag{S23}$$

where $\lambda(1)$ is obtained from the dataset, and $S_0(1)$ is calculated from equation (S20) above. Following this notation, the first element of the total activity at $t=2$ is composed of three terms:

$$\lambda(2)=S_0(2)+S_1(1)+S_2(0), \tag{S24}$$

from which the Exogenous shock $S_2(0)$ at this time is solved:

$$S_2(0)=\lambda(2)-\big(S_0(2)+S_1(1)\big), \tag{S25}$$

where $S_0(2)$ and $S_1(1)$ are obtained through equation S21 above.

Thus, in general we have:

$$S_t(0) = \lambda(t) - \sum_{g=0}^{t-1} S_g(t-g). \quad \text{(S26)}$$

In other words, assuming every new generation decays with the same decay constant as the total activity after the peak, the new contribution to the activity at time t, (or $S_t(0)$) is obtained recursively by subtracting from $\lambda(t)$ the contribution from all previous generations that are given by equation (S21), keeping track of the "local time" $(t-g)$ of the new generations.

Therefore, using equation (S26), all the information needed to extract the magnitude of the Exogenous sources is the total attendance $\lambda(t-t_c)$ vs. time, as obtained from the dataset and the observed decay constant after the activity peak.
