# Supplementary material for: Dynamical Signatures of Collective Quality Grading in a Social Activity: Attendance to Motion Pictures
Source: PLoS One. 2015 Jan 22;10(1):e0116811. doi: 10.1371/journal.pone.0116811 (PMC4303319; doi:10.1371/journal.pone.0116811)
Supplement: S3 Appendix — (PDF) [file pone.0116811.s003.pdf]

SUPPORTING INFORMATION for the paper:

Dynamical signatures of collective quality grading in a social activity: attendance to motion pictures

by Juan V. Escobar & Didier Sornette

S3 Appendix: On data filtering.

The initial data set consists of 10,000 movies that played in the US from 1970 to 2010. The data for maximum Gross per Week was inflation adjusted using the *Consumer Price Index* of the corresponding year the movie was in the theatres. Out of the total, 48.5% played for at least 4 consecutive weeks. Since fitting an exponential on only three data points yields too large an error, we impose the condition of being played for at least 4 consecutive weeks as a first filter. The distribution of the R^2 coefficients of the fits made on this selected 48.5% is presented in figure S2.

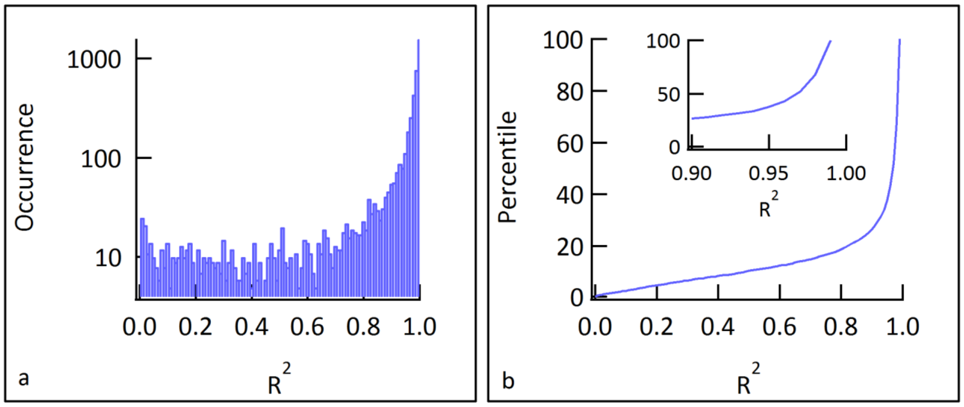

Figure S2. (a) R^2 coefficient distribution of the fits of the Gross revenue per week to equation 1. (b) Corresponding percentiles. (Inset) Close up of figure b: about 75% of the fits have an R^2>0.9

This figure evidences that, after the first filter, the fits were on average very good: at least 75% had R^2>0.9. Nevertheless, an important variable that actually determines the statistical validity of our analysis is the maximum number of theaters (Max_{th}) that played a certain movie. If Max_{th} is too small, then it is more likely that the attendance per week will be influenced by local effects. In order to determine what would be a reasonable limit on Max_{th}, we analyze in figure S3 both the average R^2 coefficient and the observed decay constant $1/\tau_0$ as a function of Max_{th}

Note in fig. S3a that the average R^2 coefficient improves drastically for movies with Max_{th}>50, after which value it remains almost constant. Furthermore, the few movies that yielded a negative decay rate belong predominantly to movies with Max_{th}<50 (fig. S3b) and, as shown in figure S3d, these negative $1/\tau_0$ values are actually associated with bad fits, and are therefore unwanted. On the other hand, we are only interested in those movies that had a large enough number of votes to allow us to calculate an average perceived quality that is representative. In this

respect, figure S3c shows the relation between Max_{th} and the average number of votes. Note that, for Max_{th}=50, this average is about 40, a number that meets our statistical requirement. Finally, we note that 50 theaters correspond to one theater per U.S. state on average.

Summarizing, setting 50 as the lower limit to Max_{th} emerges naturally because it ensures that

1) The quality of the fits is improved.
2) There is but a negligible number of movies with negative $1/\tau_0$.
3) There are enough votes to calculate a statistically significant average perceived quality.

This is the second filter we use, which makes our statistical analysis robust. Seventy percent of the movies that had gone through the first filter meet this second criterion (3,469 movies).

The distribution of R^2 coefficients of the fits to eq. 1 of the resulting dataset (the one used in this article) is presented above (figs. S4c,d) along with the distribution of the corresponding observed decay rates $1/\tau_o$ (figs. S4a,b) and the distribution of the number of votes (figs. S4e,f). As an end result, 90% of the fits have an R^2 value better than 0.98 and about 95% of the movies have at least 50 votes from which the perceived quality is obtained with confidence.

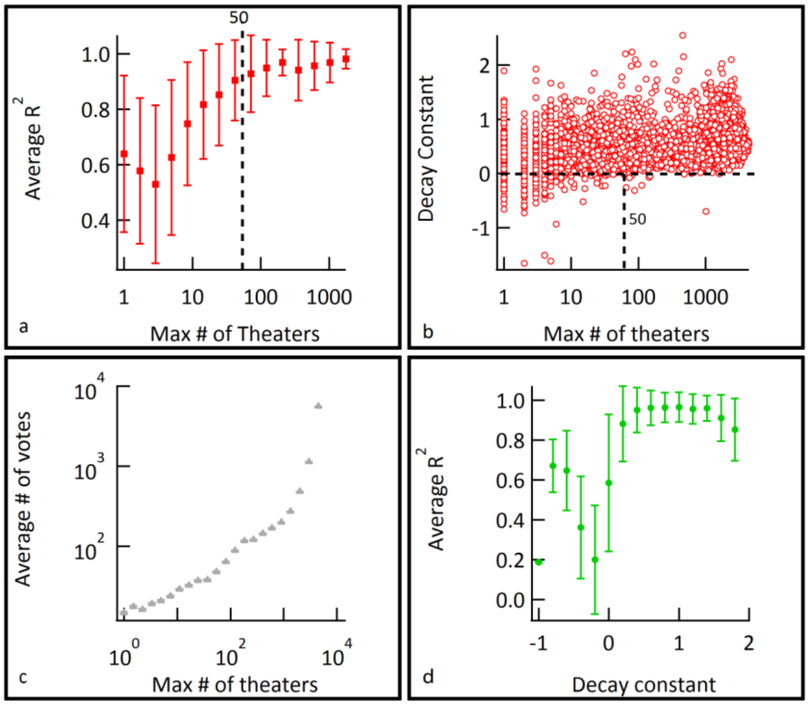

Figure S3. Data analysis after the first filter: a) Average R^2 coefficient *vs.* Max_{th} ; b) Observed $1/\tau_o$ *vs.* Max_{th} ; c) Average number of votes *vs.* Max_{th} ;; d) Average R^2 coefficient *vs.* $1/\tau_o$.

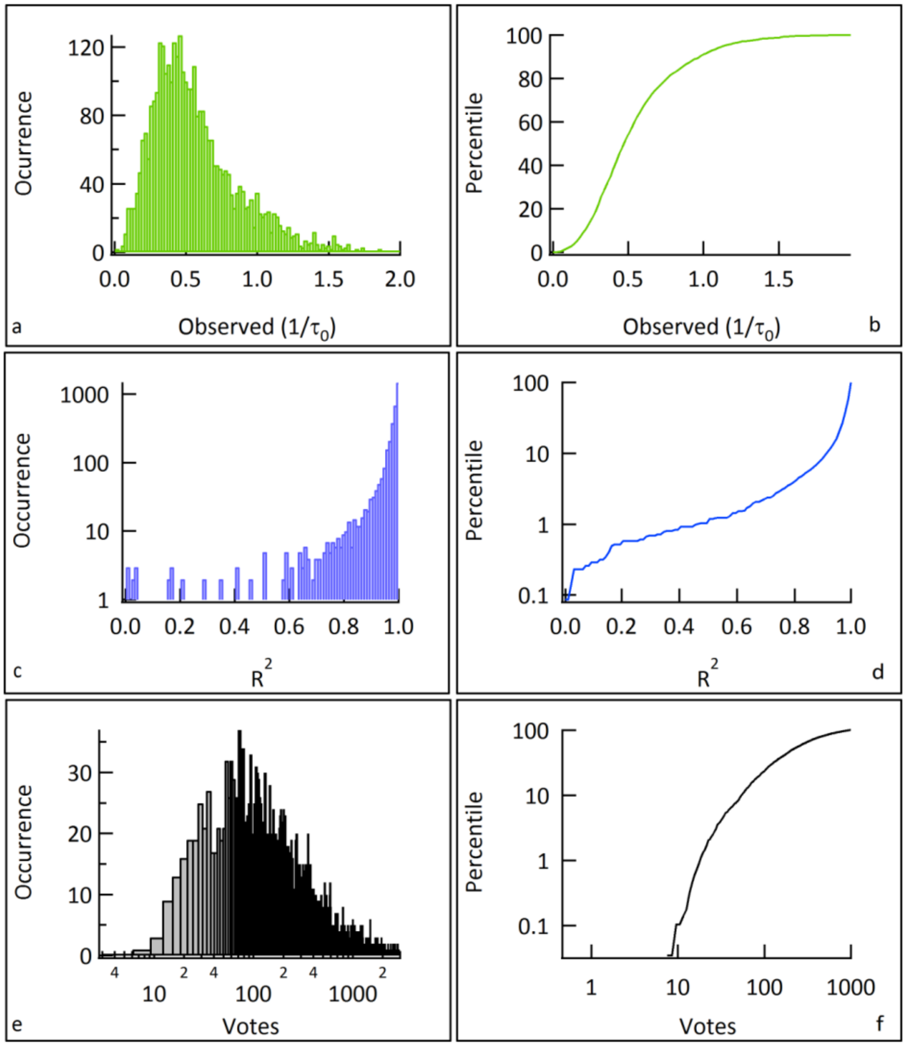

Figure S4. Data analysis of the filtered data (3469 movies). Distribution/percentiles of: (a/b) the observed $1/\tau_o$, (c/d) the R^2 coefficients and (e/f) the number of votes for each movie.
