# Supplementary material for: Dynamical Signatures of Collective Quality Grading in a Social Activity: Attendance to Motion Pictures
Source: PLoS One. 2015 Jan 22;10(1):e0116811. doi: 10.1371/journal.pone.0116811 (PMC4303319; doi:10.1371/journal.pone.0116811)
Supplement: S4 Appendix — (PDF) [file pone.0116811.s004.pdf]

SUPPORTING INFORMATION for the paper:

Dynamical signatures of collective quality grading in a social activity: attendance to motion pictures

by Juan V. Escobar & Didier Sornette

S4 Appendix: Finding the axis of symmetry

As noted in the results section, the branching ratio n *vs.* G graph of figure 3a shows not only that these variables are correlated, but also that there exists an axis of symmetry (almost the same one as the principal axis of covariation) of the data which is necessary to find if one wishes to obtain the best prediction of $n(G)$.

In order to find this axis, the following procedure is followed for a given bare kernel decay rate $1/\tau$. First, the whole dataset –the n vs. G data (fig S5a) –is rotated by an angle α, and a quantity Δy is added to the rotated G such that the mean of the resulting distribution is zero (figure S5b). The rotated n *and rotated* G *plus* Δy *are called the "transformed variables". (*Note that the "transformed Grade" axis in figure S5 is $G' \equiv G\,Cos(\,\alpha) - Tan(\alpha)\{Cos(\alpha)n + \Delta y\}$ and the transformed n is $n' = \left(\left(n_{pred} - n\right)^2 + (G'Cos(\alpha) - G)^2\right)^{1/2}$.

The difference between the rotated G and the horizontal is fit to a normal distribution (fig. S5d), and the obtained chi^2 value as a function of α is then plotted and fit to a parabola (fig. S5e). The angle that minimizes this function is then chosen. *The rotated and original values can be contrasted in figures S5a and S5b along with the corresponding distributions and fits to normal distributions in figures S5c-d.* Figures S5f and S5g show respectively the average values and corresponding standard deviation of the data on the transformed axes. Note that the standard deviation is almost constant and the average is close to zero. Thus, the proposed axis of symmetry minimizes the skewness of the data and provides the correct reference around which to obtain the averages and deviations. Note that this standard deviation is much smaller than that of the original data, as shown in the inset of figure 3c of the main text of the article. A similar result could be obtained by doing a principal component analysis that maximizes the variance of the data on the transformed axis. Here, we prefer to use as the best prediction the symmetry axis of the data because the distribution of the deviations is remarkably well fit by a Normal function (fig. S5d).

In principle, the decay rate $1/\tau$ could have been chosen to be a function of γ, but we decided to keep it constant because it provides the simplest possible model, and also due to the good results that this choice has brought in the study of other social systems.

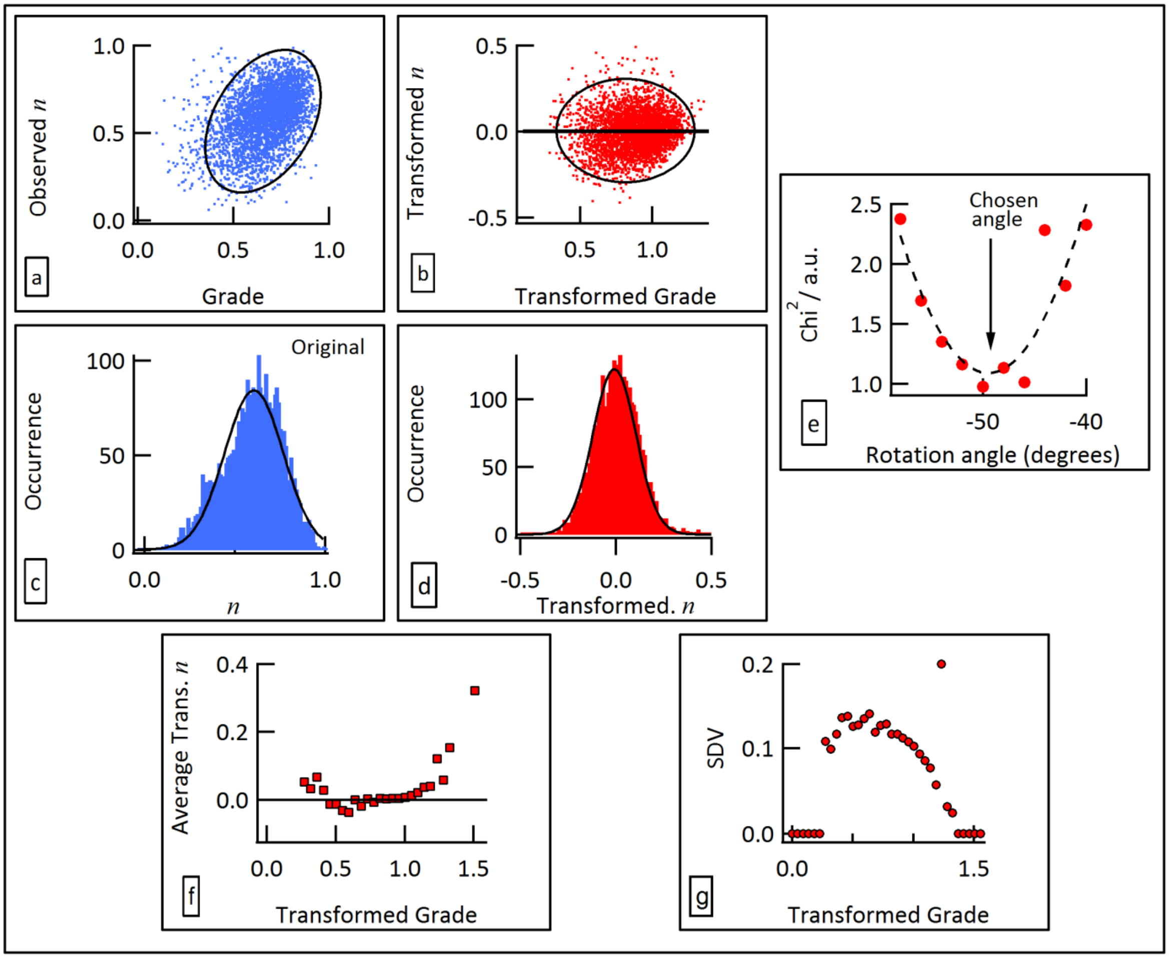

Figure S5. Determination of α and Δy: Observed (a) and transformed (b) n *vs.* G. (c-d) Corresponding distributions of n and transformed n. (e) Chi^2 vs. Rotation angle (circles) and parabolic fit. The minimum of the parabola gives the chosen angle α. (f) Average transformed n vs G., showing that the transformation has reduced the skewness and made the mean close to zero. (g) Corresponding SDV of the data points of figure ***f****. The parameters obtained from this analysis are* α=-49.5^o , Δy=0.118 and $(1/\tau) = 4$.
