# Supplementary material for: Dynamical Signatures of Collective Quality Grading in a Social Activity: Attendance to Motion Pictures
Source: PLoS One. 2015 Jan 22;10(1):e0116811. doi: 10.1371/journal.pone.0116811 (PMC4303319; doi:10.1371/journal.pone.0116811)
Supplement: S5 Appendix — (PDF) [file pone.0116811.s005.pdf]

SUPPORTING INFORMATION for the paper:

Dynamical signatures of collective quality grading in a social activity: attendance to motion pictures

by Juan V. Escobar & Didier Sornette

S5 Appendix: Total revenue vs. Grade

In reality, movies play for a finite time only (5 weeks on average) and actually, the better the movie, the longer it will play in theaters, so that eq. 11,

$$Total\ Revenue \approx \gamma \sum_{t=t_c}^{\infty} e^{-(t-t_c)/\tau_0} = \gamma\left(\frac{1}{1-e^{-1/\tau_0}}\right) = \gamma\left(\frac{1}{1-e^{-(1-\theta)/\tau}}\right) \approx \gamma\left(\frac{1}{1-e^{-(1-n)/\tau}}\right), \qquad (11,S27)$$

could in principle be improved by substituting the upper infinite limit by some function of G. However, the error obtained by using the infinity limit is less than 5%, as shown below.

There exists a relation between the number N of weeks during which a movie plays in theaters and the grade: as expected, the better the grade, the longer the movie stays in theaters. Figure S6 shows this relation obtained from the ≈3500 movies analyzed.

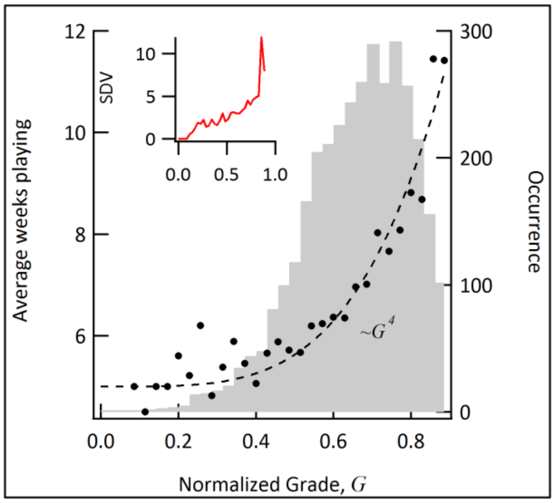

Figure S6. (left axis) Average number $\boldsymbol{N}$ of weeks of exhibition as a function of the perceived quality $\boldsymbol{G}$, which is fit (dashed line) to $\boldsymbol{N = 5 + 10G^4}$. Inset shows standard deviation, while the right axis (gray bars) depicts the occurrence of these events.

The dashed line is an approximation to the average weeks playing as a function G given by $N(G) = 5 + 10\,G^4$. Therefore, in principle the total profit can be better approximated by:

$$Total\ Revenue = \gamma\left(\sum_{t=t_c}^{N} e^{-\frac{t-t_c}{\tau_0}}\right) = \gamma\left(\sum_{t=t_c}^{(5+10G^4)} e^{-\frac{t-t_c}{\tau_0}}\right) \qquad (S28)$$

Nevertheless, the approximate revenue with the infinite limit we gave in eq. (11) happens to be a good approximation for any $N \geq 4$. To show this, consider the ratio R of the two expressions for the total revenue that we have discussed:

$$R = \left(\sum_{t=t_c}^{N} e^{-\frac{t-t_c}{\tau_0}}\right) / \left(\sum_{t=t_c}^{\infty} e^{-\frac{t-t_c}{\tau_0}}\right) \qquad \text{(S29)}$$

The following figure shows R *vs.* $1/\tau_0$ for $N = \{4,5,6,7 \text{ and } 8\}$. To prove our assertion, recall the correlation that exists between the decay constants and G. Then, Fig. S6 above implies that smaller values of $1/\tau_0$ are correlated with longer exhibition times as shown by the solid columns in the next figure.

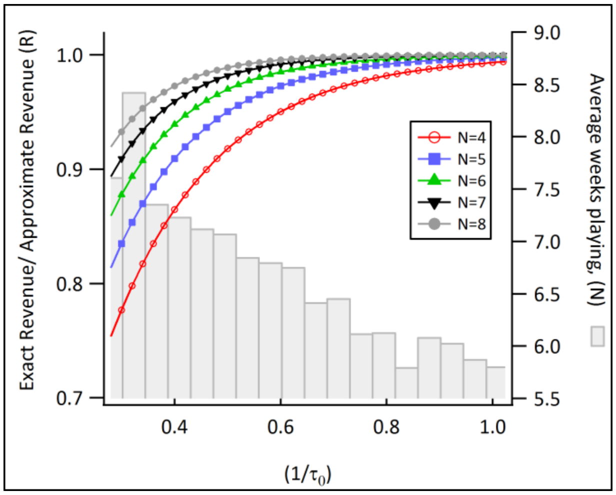

Figure S7. Comparison between total revenue obtained with infinite and finite times of exhibition. (left) Ratio $\boldsymbol{R}$ of these two quantities (eq. A29) as a function of the observed decay constant for different exhibition weeks $\boldsymbol{N}$. (right) Average $\boldsymbol{N}$ *vs.* observed decay rate $\mathbf{1}/\boldsymbol{\tau_0}$.

Note that small observed decay rates are associated with large N and *vice versa*. As a consequence, for small $1/\tau_0$<0.4, the average N is above 8, which yields a small error of about 5% (grey filled dots). On the other hand, for relatively large $1/\tau_0$>0.8 the average N is close to 5. In this case, the blue squares in that area also correspond to an error of less than 5%. The same reasoning applies for intermediate values of $(1/\tau_0)$. Therefore, the error between the exact $\gamma\left(\sum_{t=t_c}^{N} e^{-\frac{t-t_c}{\tau_0}}\right)$ and the approximate one, $\gamma\left(\sum_{t=t_c}^{\infty} e^{-\frac{t-t_c}{\tau_0}}\right)$, is always on the order of 5% for any $N \geq 4$.
