# Supplementary material for: Dynamical Signatures of Collective Quality Grading in a Social Activity: Attendance to Motion Pictures
Source: PLoS One. 2015 Jan 22;10(1):e0116811. doi: 10.1371/journal.pone.0116811 (PMC4303319; doi:10.1371/journal.pone.0116811)
Supplement: S6 Appendix — (PDF) [file pone.0116811.s006.pdf]

SUPPORTING INFORMATION for the paper:

Dynamical signatures of collective quality grading in a social activity: attendance to motion pictures

by Juan V. Escobar & Didier Sornette

S6 Appendix: Correlation between the normalized grade G used in the present paper, and the "Audience Average Rating" obtained from the website www.rottentomatoes.com.

The "Audience Average Rating" (ARR) was obtained from www.rottentomatoes.com for each one of the 3,469 movies that met both criteria of data filtering (see appendix III). The ARR could not be found for only 6 movies. These movies were removed in the analysis of this appendix.

The following figure displays G vs. ARR. There exists a positive linear correlation between these two variables, (Pearson Coefficient = 0.62). Thus, similar results to the ones presented in this paper are likely to be obtained using instead the grading info from www.rottentomatoes.com.

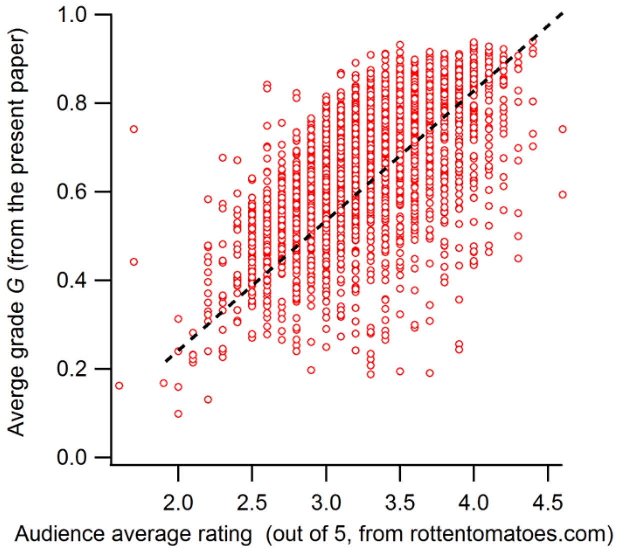

Figure S8. Linear correlation between the normalized grade G used in the present paper, and the "Audience Average Rating" obtained from the website www.rottentomatoes.com (Pearson Coefficient = 0.62).
