# Supplementary material for: Dynamical Signatures of Collective Quality Grading in a Social Activity: Attendance to Motion Pictures
Source: PLoS One. 2015 Jan 22;10(1):e0116811. doi: 10.1371/journal.pone.0116811 (PMC4303319; doi:10.1371/journal.pone.0116811)
Supplement: S7 Appendix — (PDF) [file pone.0116811.s007.pdf]

SUPPORTING INFORMATION for the paper:

Dynamical signatures of collective quality grading in a social activity: attendance to motion pictures

by Juan V. Escobar & Didier Sornette

S7 Appendix: General Trends of the data.

The nature of the data can be better visualized by plotting $1/\tau_0$ and t_c as a function of both the normalized perceived quality variable G and the average natural logarithm of γ in 3D color plots. This is displayed in figures S9a and S9b respectively, while figure S9d shows the density of occurrences. The following facts stand out from these graphs:

1. **Effect of perceived quality on the dynamics**. At first glance, it can be appreciated in fig. S9a that better grades correspond to smaller $1/\tau_0$ as corroborated by the 2D projection of fig S9a displayed in figure S9c. In other words, the better the grade, the longer is the lifetime of the movie in theatres. Note, however, that the corresponding standard deviations as presented in the inset of this figure are much larger than the standard deviation of 0.11 around the axis of symmetry presented in figure 3a of the main paper.

2. **Quality of endogenous shocks.** Movies with $G > 0.5$ present an average value of $t_c = 5$ weeks (orange and yellow squares in fig. S9b). This fact supports the intuitive notion that only movies that were perceived as "good" had their maximum activity after the second week since the opening. Furthermore, the sharp decline to zero density below $G = 0.5$ for Endogenous shocks indicates that this grade is a measure of the limit below which the social system itself considers a movie to be bad. These observations validate our choice of function G to normalize the perceived grade.

3. **Dependency of $1/\tau_0$ on Ln(γ).** Note in figure S9a that the zone composed of larger decay constants $1/\tau_0$ (shortest lived movies, yellow squares) transitions to smaller values of $1/\tau_0$ (longer lived movies, red squares) for *Ln*(γ)>15. In other words, for two movies with the same grade, a larger *Ln*(γ) results in a slower decay and a longer lifetime. As a practical consequence, if two movies are perceived as being of equal quality, the one that draws a larger audience on the opening day will end up making a lot more money by the conjunction of a larger level of attendees together with a longer lifetime.

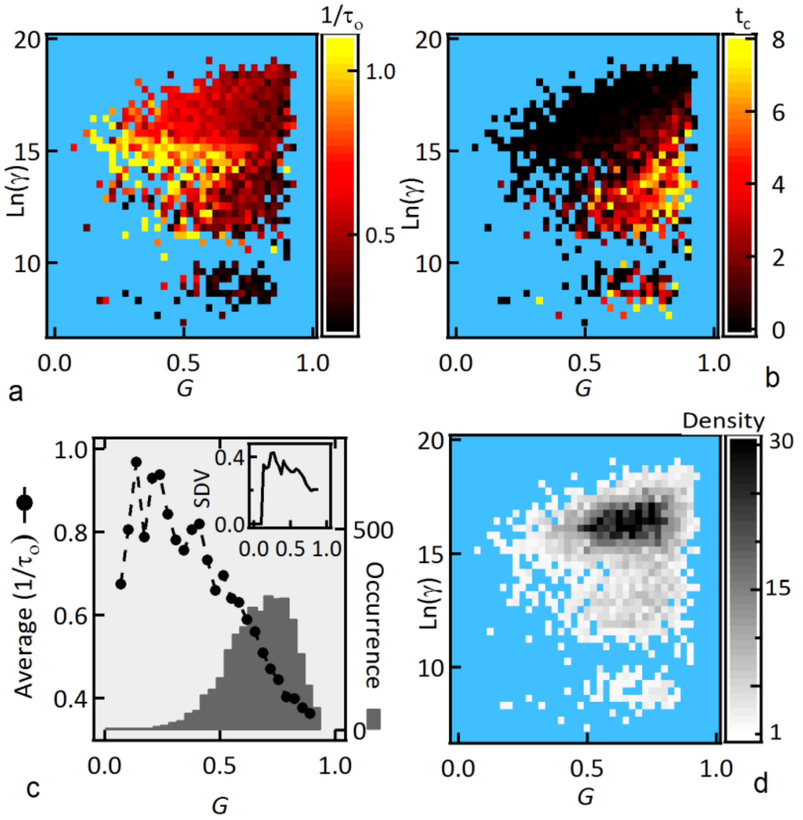

Figure S9. A first look at the data: In panels (a), (b) and (d), the magnitude of the maximum level of attendance $Ln(\gamma)$ is shown as a function of grade G for the ~3500 movies analyzed in this study. The set of occurrences is the same and what distinguishes the three panels is the color code on the right vertical axis of each panel giving the amplitude of the following variables: (a) Decay constants $1/\tau_0$ (average R^2=0.96), (b) t_c and (d) density. Panel c) shows the average decay constant as a function of the average grade (2D projection of figure S9a) showing the correlation between these variables. Inset in fig. S9c shows the corresponding standard deviation.
