# Supplementary material for: Dynamical Signatures of Collective Quality Grading in a Social Activity: Attendance to Motion Pictures
Source: PLoS One. 2015 Jan 22;10(1):e0116811. doi: 10.1371/journal.pone.0116811 (PMC4303319; doi:10.1371/journal.pone.0116811)
Supplement: S8 Appendix — (PDF) [file pone.0116811.s008.pdf]

SUPPORTING INFORMATION for the paper:

Dynamical signatures of collective quality grading in a social activity: attendance to motion pictures

by Juan V. Escobar & Didier Sornette

S8 Appendix: An example of how new theaters serve as a source of external shocks.

Figure S10 shows an example of the performance of a movie in which the collection of small external shocks $\{S_i\}$ obtained with equation 10 is assumed to be proportional to the number of new theatres $\{\kappa_i\}$ (upper panel). When the resulting sources are incorporated into equation 3, our model depicted by the blue squares in the lower panel reproduces well the actual data (gray dots). Figure 6 of the main paper confirms that, in general, the new theaters serve as sources of new shocks, and that the magnitude of these shocks S_i (in units of gross per week) may be a linear function of κ_i.

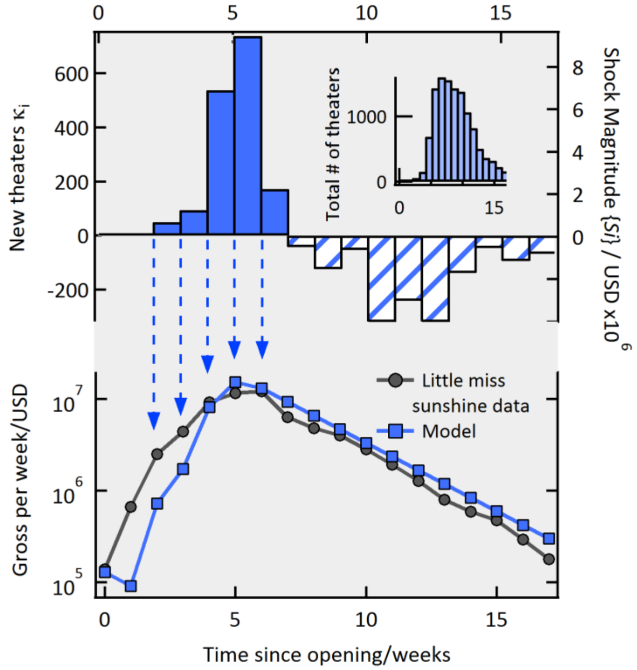

Figure S10. New theaters as sources of external shocks. New theaters that open up κ_i (Filled columns, left axis, upper graph) act as sources of external shocks S_i, (right axis, upper graph) assuming a proportionality factor of 12,800 USD/(theater x week). The blue squares and trace in the lower graph depicts the activity obtained with equation 3, while the gray dots and trace are the actual data of the gross per week for the movie *Little Miss Sunshine (2006).* The same observed decay constant $1/\tau_0$ for the relaxation process was ascribed to every new generation and subtracted from the total revenue for that week (eq. 10). On the other hand, the theaters that are being removed (striped columns top graph) do not contribute to the attendance activity. Thus, the full activity including excitation and relaxation is described by equation 3. Inset: Total number of theaters for that particular movie *vs*. time.
