# Supplementary material for: Dynamical Signatures of Collective Quality Grading in a Social Activity: Attendance to Motion Pictures
Source: PLoS One. 2015 Jan 22;10(1):e0116811. doi: 10.1371/journal.pone.0116811 (PMC4303319; doi:10.1371/journal.pone.0116811)
Supplement: S9 Appendix — (PDF) [file pone.0116811.s009.pdf]

**SUPPORTING INFORMATION for the paper:**

***Dynamical signatures of collective quality grading in a social activity: attendance to motion pictures***

**by Juan V. Escobar & Didier Sornette**

**S9 Appendix: Note on medians.**

We point out that deviations of the median values with respect to the model in fig. 6 may seem large but are actually very close to the model curve. The reason is that the averages and medians are calculated in a different reference frame (like those in figure 3a, that are calculated with respect to the axis of symmetry in the rotated frame as explained in the appendix), and then are translated to the original reference frame in such a way that they are normal to the model curve. In the case of the average branching ratio (fig 3a), only a rotation is needed because the direction of the normal vector to the symmetry axis is constant for all $G$. But the derivative of the predicted revenue becomes very large already for $G>0.5$ (fig 7), and the normal becomes almost horizontal for such values of $G$. Thus, a relatively small error on the revenue with respect to the model of about 0.3 (small because the Revenue axis goes from 0 to 10) translates into a large apparent error in figure 7 because it all goes into the horizontal ($G$) axis, which spans only the range [0,1].
