# Supplementary material for: Dynamical Signatures of Collective Quality Grading in a Social Activity: Attendance to Motion Pictures
Source: PLoS One. 2015 Jan 22;10(1):e0116811. doi: 10.1371/journal.pone.0116811 (PMC4303319; doi:10.1371/journal.pone.0116811)
Supplement: S10 Appendix — (PDF) [file pone.0116811.s010.pdf]

SUPPORTING INFORMATION for the paper:

Dynamical signatures of collective quality grading in a social activity: attendance to motion pictures

by Juan V. Escobar & Didier Sornette

S10 Appendix: Correlation between then number of movies playing, and n and n/G

We recognize that the social system under study is extremely complex, in which many factors can in principle determine the success of a movie. Some of these factors may include competition, finiteness of resources for advertising, imitation *vs.* resistance *vs*. resilience in the public, or fashion waves.

Here we briefly explore how the dynamics of the attendance is affected by the competition between movies.

To this end, we extract from the data set the number of movies playing as a function of the calendar week and, for each movie, we calculate the average total number of movies N playing during the first four weeks since its opening week. Figure S11a shows n *vs.* N for the whole Exogenous class. The negative correlation we find between these variables can be interpreted as being the result of movies competing for audiences, as would be expected. We can then ask what the effect of competition on the effectiveness of recommendations is. In figure S11b, we plot n/G *vs.* N. in which we observe again a negative correlation. In turn, this can be interpreted as a dilution of the strength of a recommendation for larger movie supplies. This can be rationalized by the fact that two acquaintances recommending strongly two different movies will have a diminishing influence per movie, given the movie goers' constraints of limited time, money or desire to be entertained. Using simultaneously the information about the expected N and G (among other factors to be potentially studied) would certainly yield better predictions for the branching ratio than the one presented in figure 3a. We stress, however, that the evidence drawn from our analysis strongly suggests the main conclusion that a cascade of recommendations and attendances takes place in this social system, as shown by their dynamic signatures in the weekly revenue. This is our main result. Further studies about the influence of other potential factors on the branching ratio would be of great interest.

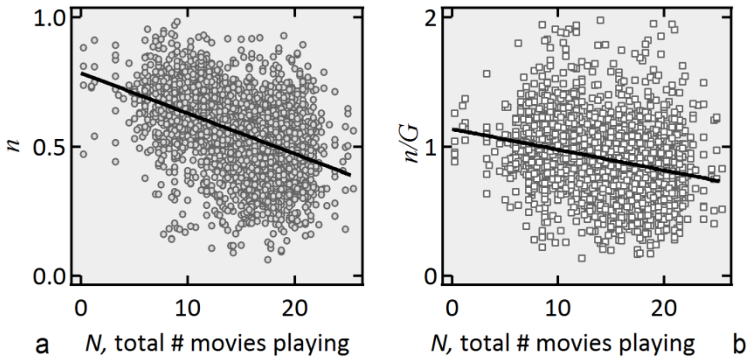

Figure S11. a) Branching ratio *n* as a function of *N*, the average total number of movies playing during the first four weeks of the target movie since its opening week. *b)* n/G *vs.* N*,* suggesting a negative effect of movie supply on the strength of recommendations. Solid lines depict the corresponding first principal component axis.
