# Supplementary material for: Dynamical Signatures of Collective Quality Grading in a Social Activity: Attendance to Motion Pictures
Source: PLoS One. 2015 Jan 22;10(1):e0116811. doi: 10.1371/journal.pone.0116811 (PMC4303319; doi:10.1371/journal.pone.0116811)
Supplement: S11 Appendix — (PDF) [file pone.0116811.s011.pdf]

SUPPORTING INFORMATION for the paper:

Dynamical signatures of collective quality grading in a social activity: attendance to motion pictures

by Juan V. Escobar & Didier Sornette

S11 Appendix: METHODS

1. Data Filtering. From the whole data set consisting of ~10,000 movies that played between 1970 to 2010, only those 3469 that played for at least 4 weeks and in at least 50 theaters were kept. In general, the fits were excellent, with about 90% of the R^2 values being higher than 0.98 (see Supporting Information for details). The data for maximum Gross per Week was inflation adjusted using the *Consumer Price Index* of the corresponding year the movie was in the theatres.

2. Finding t_c. In order to obtain the week t_c of maximum attendance for each movie, the criteria used involves finding the week of the maximum number of theaters rather than that of the maximum gross per week. The reasoning behind this is the realization that new theaters will act as sources of Exogenous shocks. Therefore, we expect that the system will relax without any further input once the number of theaters showing the movies does not increase anymore. The algorithm we implement does the following: first, the week of the maximum number of theaters is found. Then, the number of theaters from all subsequent weeks is examined, and t_c is chosen as the latest week in which at least 90% of the maximum number of theaters played that movie.

3. Criterion for deeming a shock as being Exogenous as a function of t_c. The precise day of the week of the opening for the movies analyzed varied. However, the as-found initial weekly gross was always the aggregated revenue starting from the opening day up to the following Thursday. As a result, all things being equal, a movie opening on a Thursday had a larger gross per week than one that opens on a Sunday. In order to take this fact into account, the criterion for classifying a movie as belonging to the Exogenous class was not exclusively t_c =0, but also t_c =1.

4. Fit to a decaying exponential. An algorithm designed to fit noisy data was implemented [6] that is able to signal when a change of dynamics has taken place. For a given movie that played for (L - 1) weeks after t_c, this algorithm first creates sub-sets $\{\lambda(t_c), \lambda(t_c + 1), \lambda(t_c + 2), \ldots, \lambda(t_c + n-1), \lambda(t_c + n)\}_n$ of the Gross revenue per week (or λ(t)) of increasing length n, where n ranges from $n_0 \equiv \min(10, L-1)$ up to (L-1). It then proceeds to fit each one of these subsets to a decaying exponential (eq. 1) from which it calculates the decay constant c_n and corresponding standard deviations σ_n, where the subscript n refers to the length of the subsets. For every subset, the algorithm then takes the point $\lambda(n+1)$ and evaluates the probability P_{n+1} that this point belongs to the same set of length n assuming the residuals of the fit are normally distributed with variance σ_n^2. This probability is given by $P_{n+1} = \left(1 - \frac{2}{\sqrt{\pi}} \int_0^{x_{n+1}/\sqrt{2}} e^{-t^2} dt\right)$, where $x_{n+1} \equiv \left(Dif_{n+1} / \sigma_n\right)$, and Dif_{n+1} is the difference between $\lambda(n+1)$ and the best fit to the original subset of length n. If this probability is greater than .003 (i.e., it lies within $3\sigma_n$ of the fit) then the point (n+1) is deemed as belonging to the subset of length n, and a 1 is added to total number of points i_n that will be considered as belonging to the subset. The algorithm then restarts with the point (n + 2) and continues until all new points have been assessed or until the probability that three consecutive points lie outside

of the $3\sigma_n$ limit. This happens when (P_{n+j} x P_{n+j+1} x $P_{n+j+2} < (0.003)^3$) for some point j. Once it has stopped, this algorithm yields a triad of values (i_n, c_n, σ_n) for every n, and a collection {(i_{no}, c_{no}, σ_{no}), (i_{no+1}, c_{no+1}, σ_{no+1}),...,(i_{L-1}, c_{L-1}, σ_{L-1})} for every movie. The triad with the maximum i_n is selected (i.e. the largest number of points added before finding three consecutive points that violate the $3\sigma_n$ criterion), and the corresponding decay constant of that same triad is taken as the parameter that best fits the noisy data (i.e., what we call the observed decay rate $1/\tau_0$. In case of a tie, the triad with the smallest standard deviation is chosen. The three consecutive "bad" point criterion allows the data to be naturally noisy while identifying the moment of a change of regime.

5. Grade Normalization. The grades for each movie were originally found following the American grading system consisting of a distribution of grades: *A,B,C,D* and *F*. We construct a normalized Grade *G* for every movie through the formula:

$$G \equiv \frac{1}{20}\left(\frac{10A+7B+0C-7D-10F}{A+B+C+D+F}+10\right)$$

which can rewritten as

$$G \equiv \left(0.5A' + \left(\frac{7}{20}\right)B' + (0)C' - \left(\frac{7}{20}\right)D' - 0.5F'\right) + 0.5 \ .$$

This normalization warranties that $G \in \{0,1\}$ and gives equal weight but opposite signs to the coefficients of the pairs (A',F') and (B',D'), defined as the frequencies of corresponding grades *(A, F)* and *(B, D)* respectively. In other words, the function is symmetric with respect to the relative number of votes C'. Consider the example of a movie that received votes according to the following breakdown: 100 "A"´s, 80 "B"´s, 30 "C"´s, 12 "D"´s and 4 "F"´s. For this movie, the grade would be

$$G \equiv \frac{1}{20}\left(\frac{10*100+7*80+0*30-7*12-10*4}{100+80+30+12+4}+10\right) \cong 0.81$$

Other more sophisticated non-linear normalization functions are likely to yield better correlations with the observed decay constants than those shown of fig 3a. While the original grading data can no longer be found at www.boxofficemojo.com after its merger with www.imbd.com, the "Audience Average Rating" from the popular website www.rottentomatoes.com yields a positive correlation with the normalized grade we use in the analysis of the present paper (see Supporting Information, S6 Appendix). Similar results to ours are likely to be obtained with that database.
